# A Broad Overview and Review of CRISPR-Cas Technology and Stem Cells

**DOI:** 10.1007/s40778-016-0037-5

**Published:** 2016-02-11

**Authors:** Simon N. Waddington, Riccardo Privolizzi, Rajvinder Karda, Helen C. O’Neill

**Affiliations:** 1 Gene Transfer Technology Group, Institute for Women’s Health, University College London, 86-96 Chenies Mews, London, UK; 2 Antiviral Gene Therapy Research Unit, Faculty of Health Sciences, University of the Witswatersrand, Johannesburg, South Africa; 3 Faculty of Medicine, Department of Surgery and Cancer, Imperial College London, London, UK; 4 Preimplantation Genetics and Embryology Group, Institute for Women’s Health, University College London, 86-96 Chenies Mews, London, WC1E 6HX UK

**Keywords:** Genome engineering, Gene therapy, CRISPR-Cas, iPSCs, Human Genome Editing

## Abstract

The pinnacle of four decades of research, induced pluripotent stem cells (iPSCs), and genome editing with the advent of clustered, regularly interspaced, short palindromic repeats (CRISPR) now promise to take drug development and regenerative medicine to new levels and to enable the interrogation of disease mechanisms with a hitherto unimaginable level of model fidelity. Autumn 2014 witnessed the first patient receiving iPSCs differentiated into retinal pigmented epithelium to treat macular degeneration. Technologies such as 3D bioprinting may now exploit these advances to manufacture organs in a dish. As enticing as these prospects are, these technologies demand a deeper understanding, which will lead to improvements in their safety and efficacy. For example, precise and more efficient reprogramming for iPSC production is a requisite for wider clinical adoption. Improving awareness of the roles of long non-coding RNAs (lncRNAs) and microRNAs (miRNAs) and genomic epigenetic status will contribute to the achievement of these aims. Similarly, increased efficiency, avoidance of off-target effects, and expansion of available target sequences are critical to the uptake of genome editing technology. In this review, we survey the historical development of genetic manipulation and stem cells. We explore the potential of genetic manipulation of iPSCs for in vitro disease modeling, generation of new animal models, and clinical applicability. We highlight the aspects that define CRISPR-Cas as a breakthrough technology, look at gene correction, and consider some important ethical and societal implications of this approach.

## Introduction

The time has come, it may be said,

To dream of many things;

Of genes—and life—and human cells—

Of Medicine—and kings—

Edward L Tatum, Perspectives in biology and medicine 1966 [1]

**Fig. 1:**
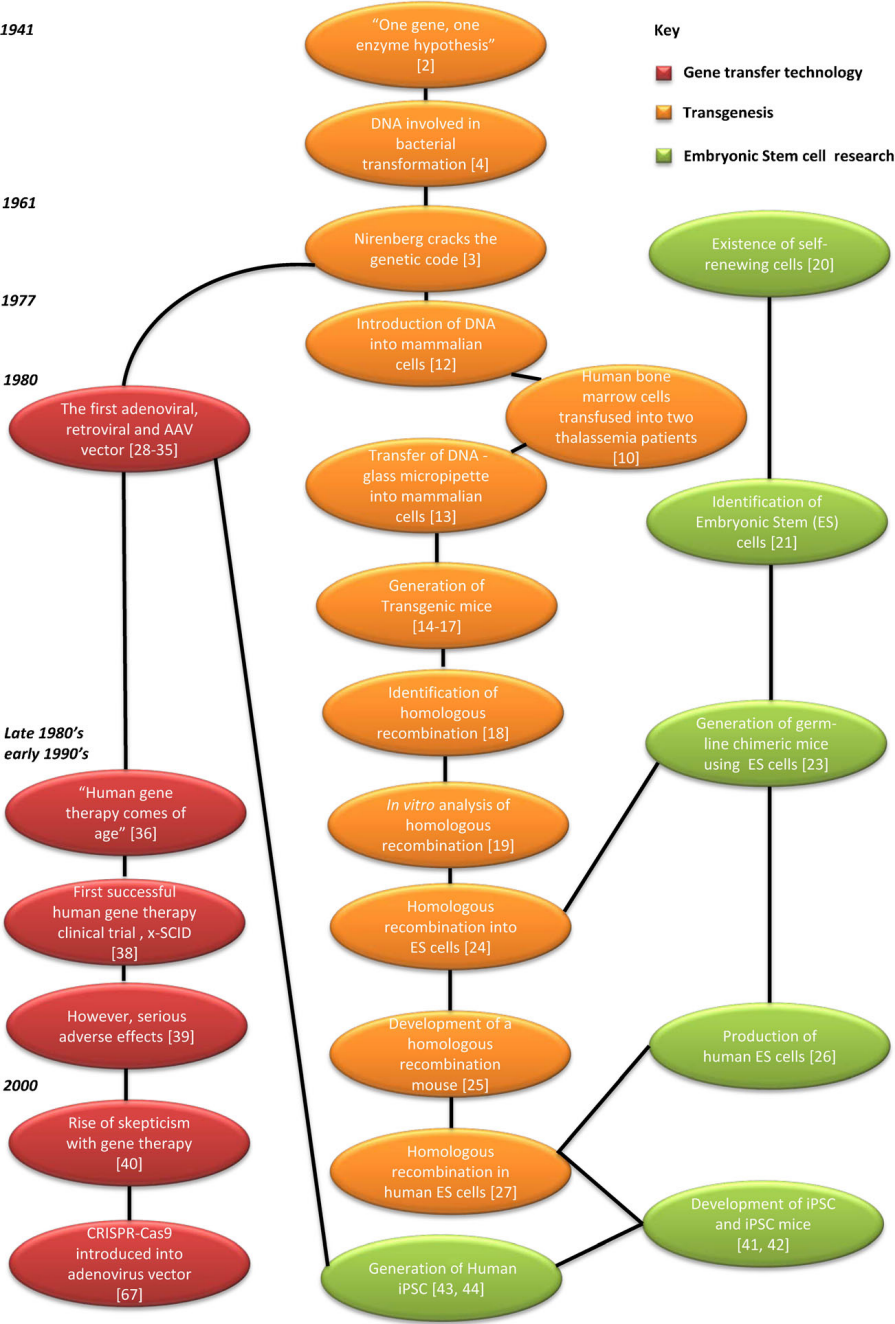
A flow diagram illustrating the generation of stem cell research, transgenesis, genetic engineering, and gene transfer technology. This time line is split into three major technology streams which converged to establish the use of CRISPR-Cas technology and stem cells

In 2015, Science Magazine recognized “CRISPR genome editing” as breakthrough of the year. Six years earlier, the “Return of Gene Therapy” was recognized as a runner up, and in 2008, the winning accolade was awarded to “Cellular Reprogramming.” These combined technologies are not only showing great promise but also are actually starting to deliver on great promises. Yet they are not new; their births can each be traced back several decades. To provide context on today’s achievements, and perhaps to predict the forthcoming trajectory, it is worth considering their developmental timelines (summarized in Fig. 1).

In 1941, Edward Tatum and George Beadle used the fungus *Neurospora* to arrive at their paradigmatic “one gene one enzyme” hypothesis [2]; for this, they were jointly awarded the Nobel Prize in Physiology with Joshua Lederberg in 1958. In 1961, Marshall Nirenberg and Heinrich Matthei began to decipher the relationship between codons and amino acids [3]. Mindful of these advances, Edward Tatum tentatively predicted regenerative medicine and gene therapy:

Hence, it can be suggested that the first successful genetic engineering will be done with the patient’s own cells, for example, liver cells, grown in culture. The desired new gene will be introduced, by directed mutation, from normal cells of another donor by transduction or by direct DNA transfer. The rare cell with the desired change will then be selected, grown into a mass culture, and reimplanted in the patient’s liver.

Precedents for the introduction or transfer of genes from one cell to another exist in microbial systems and are now being tried with mammalian cells in culture…If this can be done successfully … it will facilitate the development of a mammalian somatic cell genetics. It will also bring us considerably closer to successful genetic engineering [1].

The development of cell lines in the early 1960s tested the notion that foreign DNA could be permanently and stably introduced into mammalian cells with both functionality and heritability. Conceptually, these studies were derived from work on pneumococci carried out by Avery, MacLeod, and McCarty two decades earlier, showing that DNA was involved in bacterial transformation [4].

It became clear by the mid-late 1960s that genetic transformation by exogenous DNA was more efficient than yet suspected. Work on viral DNA in SV40-transformed cells showed that viral genomes also had the capacity to be covalently and stably transformed into target cells [5, 6]. These experiments preceded the era of recombinant DNA, so it was unclear how viruses may be modified to express or incorporate foreign genes and be used as therapeutic agents. Rogers and co-workers were the first to use viruses to transfer foreign genes into human cells [7]. However, their treatment of two girls with hyperargininemia failed to achieve any therapeutic outcome [8].

At this time, the first tools for recombinant DNA engineering were being assembled; the advent of mammalian genetic modification was close, and scientists recognized the need to define both safety and regulation [9]. A prime example of the need to enforce this regulation was the Cline experiment in 1979, which led to the introduction of the human globin gene into murine bone marrow cells. This partially repopulated the bone marrow of irradiated mice with the genetically modified marrow cells. This technique, using calcium phosphate transfection, was prematurely applied on human bone marrow cells and transfused into two thalassemia patients in 1980. The treatment was not successful and was done without the approval of the FDA [10, 11].

Michael Wigler and colleagues (among others) had previously used calcium phosphate transfection to deliver a fragment of viral DNA containing the thymidine kinase gene into a mouse cell line. However, this was woefully inefficient, with successful transfection in fewer than 1 in 100,000 cells [12]. By direct intra-nuclear injection of DNA using a glass micropipette, Mario Capecchi was able to improve hugely upon the efficiency, achieving a success rate of 1 in 5 cells [13]. This set the scene for the generation of transgenic mice, which was achieved by four different groups within 2 years [14–17]. One of these studies demonstrated the insertion of the rabbit β-globin gene and germ line transmission into the resulting mouse strain [16]. This technology provided no ability to target the genomic insertions, which remained completely random. However, Capecchi’s team noted that inserted DNA often assumed a concatemeric form, concluding this may be a product of homologous recombination [18]. This hinted at the prospect that it might be possible to target exogenous DNA to specific genomic loci, which is precisely what Oliver Smithies’ team achieved in 1988. They flanked the prospective DNA insert with sequences homologous to the human β-globin locus and used electroporation to deliver this to a hybrid human cell, observing specific insertion into this locus [19].

In 1961, the same year that Nirenberg and Matthei were decrypting codon usage, James Till and Ernest McCulloch first demonstrated the existence of multipotent stem cells: bone marrow-derived cells capable of clonal expansion, colony formation, and self-renewal [20]. Two decades later, pioneering work by Martin Evans and Matthew Kaufman revealed that early mouse embryos contain pluripotent cells—cells that have the ability to become any cell in the body (except the placenta). This so-called cell potency was defined when they were transplanted into blastocysts of mice of a different strain and produced chimeric animals. By meticulous control of isolation timing and culture conditions, they were able to maintain these cells for the first time and observe in vitro differentiation and teratoma formation when implanted into immune-deficient mice [21]. The formation of a teratoma, which contains multiple tissue components, signified the ability of these cells to develop into more than one germ layer. Shortly afterwards, Gail Martin generated similar data using a teratocarcinoma feeder cell line to maintain these stem cells. She gave these cells the name with which we are so familiar now; “embryonic stem cells” [22]. In 1984, Martin Evans and colleagues demonstrated that they could generate germ line chimeric mice by the introduction of embryonic stem cells, from established lines, into mouse blastocysts before reimplantation [23].

The close of the 1980s saw accomplishment of two major goals; firstly, Kirk Thomas and Mario Capecchi described site-directed mutagenesis of the hypoxanthine phosphoribosyl transferase gene in mouse embryonic stem cells using electroporation and homologous recombination [24]. Secondly, in 1989, Smithies and team took this technology to a natural conclusion, by generating germ line chimeric mice derived from embryonic stem cells which had undergone site-directed mutagenesis using homologous recombination [25]. For a decade, the application of this technology to generate mouse models of human disease was embraced enthusiastically by the biomedical research community. Then, in 1998, the potential of this technology was taken to a new level, when James Thompson and colleagues generated the first human embryonic stem cell lines from blastocysts. These could be maintained for months in an undifferentiated state using a mouse embryonic fibroblast feeder line but were capable of generating cells of endodermal, mesodermal, and ectodermal lineages with a teratoma assay [26]. Five years later, James Thompson and Thomas Zwaka demonstrated site-directed mutagenesis of human embryonic stem cells using homologous recombination and electroporation [27].

One major technological stream missing from this narrative, so far, has been that of viral vector-based gene transfer technology. More than 15 years after Edward Tatum had suggested that viruses could be used to deliver genetic material for therapeutic benefit [1], three quite different virus-based vectors were being developed—those based on adenoviruses, retroviruses and adeno-associated viruses (AAVs). Four groups were involved in the construction of retroviral vectors. They demonstrated gene delivery and genomic integration into mammalian cells at efficiency vastly superior to non-viral methods, such as calcium phosphate transfection and glass pipette microinjection [28–31]. Separately, groups were developing adenovirus-based vectors; these vectors showed promise for highly efficient delivery, the capacity for delivering longer DNA sequences, and strong expression of the desired protein [32–34]. In addition, Paul Hermonat and Nicholas Muzyczka developed vectors based upon AAV, which served as tools not only for the study of gene function and regulation, but became invaluable gene therapy vectors [35]. By 1992, numerous studies had demonstrated the delivery of therapeutically relevant human genes to rodent somatic tissues and human gene therapy trials were being initiated. A review article in the journal *Nature* entitled “Human gene therapy comes of age” typified the overweening enthusiasm of the field [36] and some advised against hyperbole, which risked damaging the entire discipline [37].

It was 2002 before the results of the first successful human gene therapy trial were published. Infants with X-linked severe combined immunodeficiency (SCID) received autologous hematopoietic stem cell transplants; these cells had been transduced with a retrovirus vector carrying functional common gamma chain gene. Reconstitution of immune function was reported in four of the five patients [38]. However, the following year, serious adverse events of leukemogenesis arising from this gene therapy were reported [39]; the field was being viewed with increasing skepticism. Therapeutic gene delivery required more preclinical work on vector safety, targeting, and efficacy, and for the remainder of the decade, this was performed in adverse financial conditions, as grant funding and industrial recruitment flattened [40].

However, as a fundamental research tool, retrovirus vectors were soon to prove their immense utility. A retrovirus library of 24 transcription factor genes was used to transduce mouse embryonic fibroblasts; these genes were each selected as playing a potential role in stem cell pluripotency. It is testament to the transduction efficiency of this vector that simultaneous delivery of 24 separate genes was feasible. By withdrawing or combining different factors, four of these were found necessary and sufficient to revert mouse embryonic fibroblasts to a pluripotent stem cell state. These were Oct3/4, Sox2, c-Myc, and Klf4, also known as the Yamanaka factors [41]. Generation of the resulting induced pluripotent stem cells (iPSCs) won, for Shinya Yamanaka, a share of the 2012 Nobel Prize in Physiology or Medicine. A year later, Yamanaka and colleagues demonstrated that germ line transgenic mice could be generated from iPSCs [42]. Both his team [43] and that of James Thomson [44] proceeded to generate iPSCs from human fibroblasts. In 2014, the first patient received autologous iPSCs, differentiated into retinal pigmented epithelium, to treat age-related macular degeneration. Numerous other clinical trials using embryonic stem cells were becoming available from 2010 onwards (reviewed in [45••]). The need to address the reproducibility and scaling difficulties in iPSC production was recognized by Daniel Paull and colleagues who developed a robotic system for derivation, characterization, and differentiation of iPSCs [46]. The automation of cell production has advanced even further as seen with the recent production of bioprinted mini-livers from iPSCs [47].

Stem cell development has seen continuous growth and improvement since its inception. Originally, iPSCs were generated by infecting cells with a cocktail of retroviral vectors; since these vectors integrate into the host genome, persistent expression of these factors and insertional mutagenesis increase the risk of oncogenesis. Therefore, the means of expressing reprogramming factors for sufficient duration, in the correct sequence, and in optimal amounts, are being developed. For example, Warren and colleagues designed synthetic mRNA to deliver reprogramming factors transiently yet efficiently [48]. Other strategies are reviewed by Singh and co-workers [49]. It has been shown from single-cell analyses that reprogramming consists of early stochastic and subsequent hierarchical events [50]. More recent studies have delineated the epigenetic changes over the course of reprogramming [51]; the dynamics of long non-coding RNA (lncRNA) expression [52] and the interaction between microRNAs (miRNAs) and the core reprogramming factors [53]. These, and other such studies, will be valuable in informing how to maximize the efficiency and safety of reprogramming.

Concurrent with these tremendous advances in stem cell biology, gene therapy researchers were investigating multiple strategies to improve vector safety. As with the X-SCID trial, which led to leukemia, a second trial for Wiskott-Aldrich syndrome also caused leukemia in seven out of ten patients. Both trials availed of gamma retrovirus vectors, in this case for ex vivo hematopoietic stem cell gene therapy [54]. Gamma retroviral vectors integrate in a semi-random fashion, and the expression cassette can affect genes proximal to the insertion sites, likely accounting for the leukemogenesis.

These previous efforts focused on the incorporation of DNA via viral or vector-based delivery methods in order to express a missing protein where the endogenous genome fails to do so. In recent years, an increased understanding of nuclease function has enabled more direct DNA editing. These methods aim to restore normal gene function in situ, which reduces the risks associated with random integration, as genes are controlled using endogenous regulatory elements [55].

**Fig. 2:**
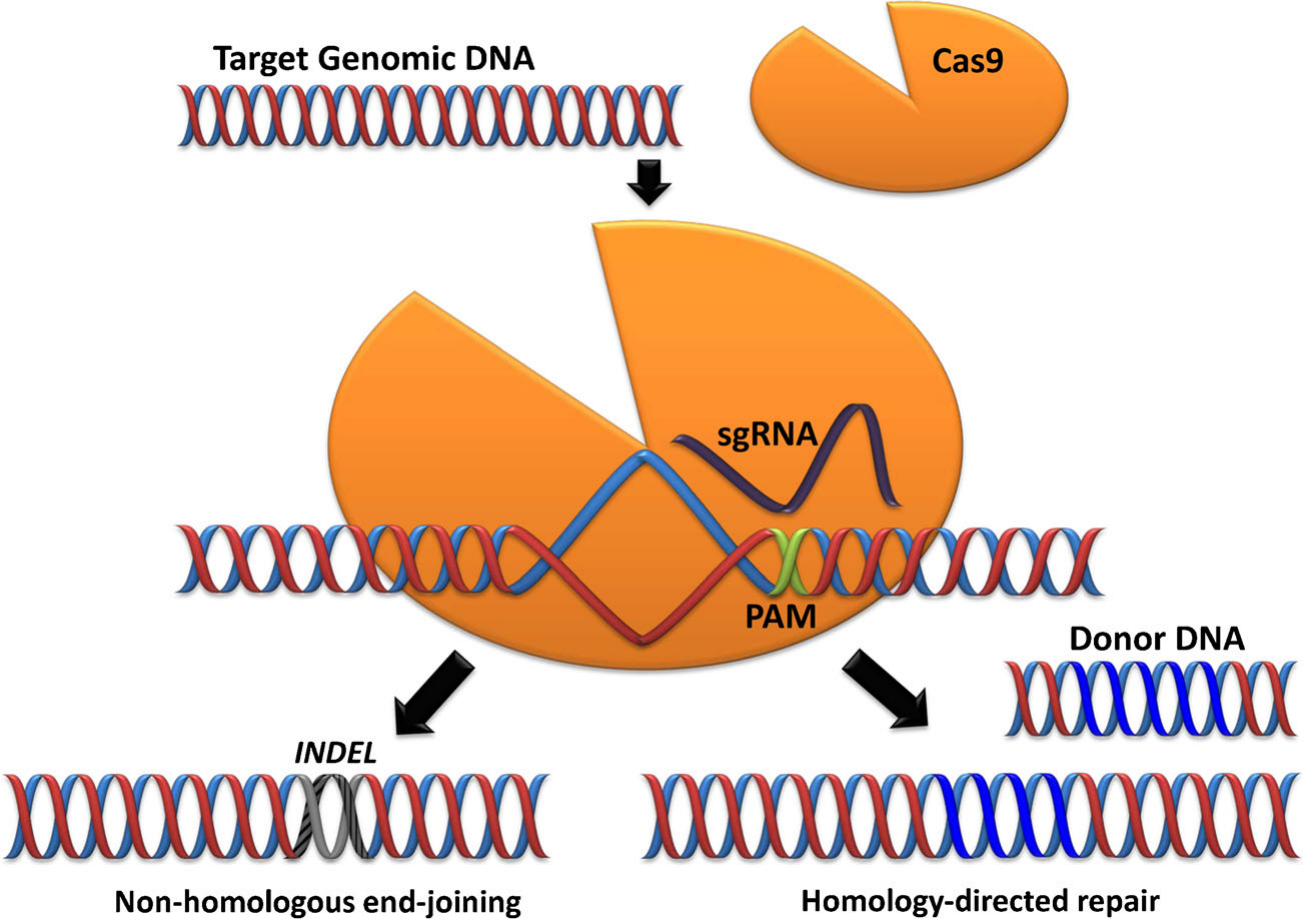
An illustration of genome editing with CRISPR-Cas9. The knock-out approach results in a loss of function of the target DNA double strand breaks by non-homologous end-joining. The knock-in results in an insertion at the repair site which exploits endogenous homology-directed repair

Crucially, targeted genome editing requires the double-stranded cleavage of DNA at the genomic locus to be modified. These double-stranded breaks (DSBs) are induced by nucleases and can be repaired by one of two mechanisms conserved across multiple organisms and cell types (Fig. 2): non-homologous end-joining (NHEJ) and homology-directed repair (HDR) [56].

Historically, gene-specific targeting has been limited to mouse embryonic stem cells. Since the discovery of iPSCs, many advances have been made in the field, with the successful differentiation into several specific cell types and establishment of patient-derived disease models [41, 57]. Establishment of a specific mutation/disease model has, until recently, relied upon traditionally low efficiency homologous recombination protocols or upon RNA interference (RNAi) (Table 1). Platforms such as meganucleases, zinc finger nucleases (ZFNs), and transcription activator-like effector nucleases (TALENs) have since been developed. These rely on protein-based systems for nuclease-directed DSBs, effectively inducing breaks that stimulate NHEJ or HDR at the specified genomic locations [58]. Such developments have permitted efficient genome editing in transformed and primary cells that were previously thought to be out of the scope of such genetic manipulation [59]. Indeed, TALENs have been used to efficiently generate mutant alleles in human pluripotent stem cells (hPSCs) of 15 different genes, as a means of performing disease modeling [60].

**Table 1:** Glossary of terms

AAV (adeno-associated virus)	A viral vector system used for gene delivery.
Chimera	A single organism composed of cells from different zygotes.
Germ line therapy	Insertion of DNA into germ line cells (egg or sperm) so that the offspring will have the inserted gene.
gRNA	Guide RNA.
Hematopoietic stem cells	Unspecialized precursor cells that will develop into mature blood cells.
Pluripotent stem cells	Stem cells that can become all cell types found in an implanted embryo, fetus, or developed organism (excluding trophoblast and placenta).
Recombinant DNA	A novel DNA sequence formed by the joining, usually in vitro, of two non-homologous DNA molecules.
Retroviral vector	A disabled RNA virus in which the viral genes have been replaced with engineered sequences.
RuvC	An endonuclease domain named for an *E. coli* protein involved in DNA repair.
sgRNA	Single guide RNA.
Stem cells	Cells with the ability to divide for indefinite periods in culture and to give rise to specialized cells.
tracrRNA, trRNA	Trans-activating crRNA.

While these site-specific nuclease technologies have made important advances in gene therapy, each has its own set of associated advantages and disadvantages, such as cost and difficulty of synthesis [61]. The CRISPR-Cas (short for clustered, regularly interspaced, short palindromic repeats/CRISPR-associated) technology has gained wide success in the global scientific community, where its power as “molecular scissors” has revolutionized the prospects of genome editing [62, 63]. Since 2012, the CRISPR-Cas system has shown potential to satisfy the shortcomings of its predecessors by adapting a naturally occurring mechanism from prokaryotic to eukaryotic systems [64–67]. The combination of the two powerful technologies of iPSCs and CRISPR-Cas is beginning to revolutionize genetic research and boost the field of precision medicine.

In considering the convergent stories of stem cell research, transgenesis, genetic engineering, and gene transfer technology, several trends emerge: (i) Each intersection of these technologies has allowed the generation of models with increasing fidelity to the human disease; this has proved valuable both in interrogating the underlying pathology and in the development and testing of therapies. (ii) Genetic engineering has improved by many orders of magnitude, in both efficiency and precision. (iii) These technologies have transitioned from being tools of basic research to having actual, or at least potential, clinical application. (iv) Improving the safety of these tools, in the laboratory and more recently in the clinic, has remained an enduring imperative.

### CRISPR/Cas Machinery and Mechanisms of Action

CRISPR systems, together with cas genes, are highly diverse mechanisms of adaptable immunity used by many bacteria and archaea to protect themselves from invading viruses, plasmids, and other foreign nucleic acids [68–71]. CRISPRs consist of a succession of highly conserved short repeated sequences (23–44 bp in length) separated by similarly sized “spacers.” These spacers are unique sequences usually originating from phage or plasmid DNA [72]. These adaptive systems can learn to recognize specific features of invading pathogens. The addition of these motifs to the host genome allows for the recognition and destruction of subsequent invasions from genetically similar pathogens. First observed in *Escherichia coli* in 1980, CRISPR loci have now been found in 84 % of sequenced archaeal genomes and approximately 45 % of bacterial genomes [73, 74]. Their function was confirmed in *Streptococcus thermophilus* in 2007, with the demonstration that resistance against a bacteriophage could be acquired by the integration of virus fragments into the CRISPR locus [68].

Adjacent to the CRISPRs are a set of CRISPR-associated (cas) genes that code for proteins essential for CRISPR activity. Comparative genomics of bacterial and archaeal genomes have suggested upwards of 45 cas gene families. The only two of these genes present in all 45 families are cas1 and cas2; both of which are involved in spacer acquisition [75, 76]. There are three major types of CRISPR-Cas systems (type I, type II, and type III), which can be further divided into ten different subtypes. Each class contains different sets of genes, repeat patterns, and species ranges.

The CRISPR/Cas system of immunity is comprised of three steps; adaptation, expression, and interference. The adaption stage involves the recognition and cleavage of a protospacer from invading DNA by the cas genes. The subsequent insertion (acquisition) of foreign DNA (spacers) into the CRISPR locus is also referred to as spacer acquisition or immunization. The expression stage refers to the expression of relevant cas genes and their proteins leading to the transcription of the CRISPR array into a long RNA molecule called the precursor CRISPR RNA (pre-crRNA). Cas proteins and other accessory factors then process this further into short mature crRNA. In the final interference stage, this mature crRNA and other cas proteins recognize foreign nucleic acid and destroy it. This is also referred to as the immunity stage, which these mechanisms mimic [77].

Both the expression and interference stage occur differently in each of the CRISPR systems. In type I systems, Cas6e/Cas6f cut at the junction of single-stranded RNA (ssRNA) and double-stranded RNA (dsRNA) formed by hairpin loops. Trans-activating (tracr) RNAs are used in type II systems to form dsRNA, cleaved by Cas9 and RNaseIII. Type III systems use a Cas6 homolog in the direct repeat for cleavage and do not require hairpin loops [74].

With each integration of invading DNA, spacer repeat units are formed. The preference of the host to recognize and create spacer precursors (protospacers) from specific sequences along the invading genome is determined by the protospacer adjacent motifs (PAMs) [78]. PAMs are short DNA sequences (3–5 bp) that differ between the variants of CRISPR and have been shown to be important for acquisition in type I and type II, but not type III systems [79]. The process of spacer acquisition occurs in a directional manner whereby new spacers are preferentially added at one side of the CRISPR (the leader sequence) [80, 81]. The leader sequence contains promoter elements, binding sites, and elements important for spacer integration. The cumulative addition of spacers containing foreign nucleotide sequences therefore acts as a chronological record of the ancestral viral invasions since protection is then inherited by the offspring [82].

A general theme can be followed across all three systems of CRISPR-mediated immunity. In all systems, the CRISPR locus is transcribed to generate a RNA-guide protein, Cas ribonucleases process the RNA guide to form a CRISPR ribonucleoprotein (crRNP) complex. This leads to the formation of a long primary transcript, known as the pre-crRNA, which can contain secondary structures, called hairpins, if palindromic sequences exist within the CRISPR sequence. These pre-crRNA sequences are then processed into smaller units, which correspond to the spacer and repeats regions [83].

In August 2012, Jennifer Doudna and Emmanuelle Charpentier co-authored a key study demonstrating the technical potential of CRISPR-Cas to cut and splice genes with extreme ease and efficiency [84]. Due to its high degree of fidelity and comparatively simple construction, CRISPR-Cas is now widely used in genome editing. CRISPR-Cas genome editing is a type II CRISPR system; this system includes Cas9, crRNA, trans-activating crRNA (tracrRNA), and two template options for DNA repair; non-homologous end joining (NHEJ) or homology directed repair (HDR). Two nuclease domains confer cleavage ability to Cas9: the HNH domain cleaves the complementary DNA strand and the RuvC-like domain cleaves the non-complementary strand [77]. A simple illustration of this is provided in Fig. 2.

To date, every facet of the CRISPR-Cas system has been altered and improved in terms of its technical application. Starting with improved endonuclease function, an alternative to Cas9, called Cpf1, was discovered in the bacterial genera *Prevotella* and *Francisella*. Interestingly, Cpf1 is a single RNA-guided enzyme that does not require tracrRNA and generates staggered DSBs with a 4–5-nt overhang distal to a 5′ T-rich PAM [85••]. Firstly, Cas9 in its natural form requires two RNAs and generates cleavage products with blunt ends, which are less easy to work with, as DNA sequences could insert in either end. Cpf1, however, makes staggered cuts that generate a 5′ overhang, which improves the precision of DNA insertions. Secondly, unlike Cas9, Cpf1 cuts at a site distal to the gene, preserving the seed region. This is essential for target recognition if future editing is required. Thirdly, because Cpf1 is smaller and does not require a tracrRNA, it may be easier to deliver to cells.

### Application of CRISPR-Cas in Genome Editing

The therapeutic potential of CRISPR-Cas has already been demonstrated in many instances. Cas9 has been applied immunologically as an antimicrobial agent and has been developed to specifically target antibiotic resistant in highly virulent strains of bacteria [77, 86]. Gene therapy applications have also been demonstrated for monogenic diseases. Cells from human patients with cystic fibrosis showed functional repair of the *CFTR* gene in vitro in cultured intestinal stem cell organoids using CRISPR-Cas [86]. The defective gene causing hereditary tyrosinemia was corrected in mice after hydrodynamic injection of CRISPR components. This led to an expansion of mutation-corrected hepatocytes in vivo and resulted in a rescued phenotype in adult mice [87]. Advancing from therapeutic treatment, as described, to preventative techniques, muscular dystrophy was prevented when germ line mediated editing of mice with Duchenne muscular was carried out [88].

The treatment of viral infections such as HIV and hepatitis B [89] has also been demonstrated using Cas9. In the first instance, iPSCs were generated, and through genome editing, were made to be homozygous for a mutation, which confers HIV-1 resistance. In this study, wild-type iPSCs were modified using TALENS and CRISPR-Cas [90, 91••].

An important milestone, was the first genetic modifications to be carried out in primate embryos. Here, CRISPR was carried out in one-cell embryos to successfully generate modified cynomolgus monkeys [92]. This is the closest of the animal models in similarity to humans and can give evidence for how the system might behave in human embryos. This could be used to potentially prevent non-complex hereditary diseases, such as single gene defects. However, without full predictability of the off-target effects, germ line gene editing remains ethically and scientifically unsafe.

In terms of delivery, the CRISPR-Cas system has been directly applied to human cells by co-delivery of plasmids that encode Cas9 expression together with the necessary crRNA components [64, 93]. Recent identification of smaller Cas proteins may enable and enhance the combination of this technology with vectors that have gained increasing success for their safety profile and efficiency, such as AAV vectors [94]. Due to their relatively low immunogenicity, AAVs are commonly chosen for in vivo gene delivery for in somatic gene therapy [95].

### Advantages, Disadvantages, and Potential Applications

The main advantages of the CRISPR-Cas system are its ability to genetically modify an organism without leaving any foreign DNA behind and its versatility and simplicity of programming. Unlike the reprogramming of its predecessors, ZFNs and TALENs, which require editing of DNA-interacting domains located at different sites on the DNA-binding scaffolds, CRISPR-Cas systems changes are only executed on the recombinant RNA sequences [62, 96]. Ease of use, low cost, high speed, multiplexing potential, and equal or higher specific DNA targeting ability have secured its popularity and success across the global scientific community [65, 96, 97]. Gupta and colleagues provide a useful review comparing ZFN, TALEN, and CRISPR-Cas technologies [98].

Nevertheless, as for many other emerging technologies, delivery of the CRISPR-Cas components to the target cells remains one of the main issues [63]. The limited DNA packaging capacity of AAV vectors, however, is being addressed with the development of shorter gRNA-coding sequences and the identification of smaller Cas endonucleases, as mentioned above. A second problem concerns the number of programmable bases in Cas9, which is limited to 20 and whose specificity is subject to the PAM sequence’s position: if this is not within ten bases from the base target, the targeting frequency is greatly diminished [63, 96]. This issue is being addressed by extending the PAM preferences of Cas9 and identifying new CRISPR endonucleases [85••, 99]. The concerns over Cas9 re-cutting after successful introduction of the desired modification may be solved by exploitation of synthetic CRISPR RNAs (scrRNAs): these offer controlled silencing options through natural decay of the scrRNA itself, injection of a “sponge” sequence complementary to the scrRNA or tracrRNA, or injection of another scrRNA directed against the Cas9 gene [97]. Principle concerns that remain are the insufficient target specificity and the potential for off-target events, which are difficult to prevent and may be undetectable for current low-cost broadly used tools as well as for more expensive and less accessible whole-genome sequencing [100••]. In particular, an emergence of tools for the prediction of off-target events and gRNA design has ensued—these are reviewed by Graham and Root [87]. The latest development of a modified Cas9 variant shows remarkably fewer off-target effects while retaining full site-specific activity and illustrates the ample scope for the optimization of the system. Here, use  of a SpCas9-HF1 (for high-fidelity) variant resulted in the occurrence of off-target events undetectable by genome-wide break capture and targeted sequencing methods [101].

Besides these practical aspects, the major drawback the CRISPR-Cas system faces is restrictive legislation. The power of this gene-editing tool has caused concerns to wider society, due to the potential for irrevocable alteration of future generations, if used in germ line modifications.

### Ethical Considerations and Conclusions

Concerns about the genetic alteration of the human species from both the scientific and lay community started to rise slowly but progressively after the advent of recombinant DNA technology [102]. The call for the recent International Summit of Genome Editing in Washington 2015 echoes that of the first Asilomar conference in 1975. Both called for a moratorium on experiments due to fear that the technologies would be used for experiments which lift the ethical threshold with the potential to alter human evolution.

With the rapid advancement of CRISPR-Cas systems towards application the for treatment of diseases such as Duchenne muscular dystrophy, hemoglobinopathies, Leber congenital amaurosis, and HIV infection, ethical implications are now being discussed [63]. In particular, the first report on the use of CRISPR-Cas9 on human tripronuclear zygotes was published in 2015. These zygotes have one oocyte nucleus and two sperm nuclei and are therefore unable to develop into viable embryos. It was this work that prompted a worldwide moratorium by both biologists and ethicists on human germ line genetic engineering [103]. Although this study showed low homologous recombination events of the human β-globin (*HBB*) gene, for which the CRISPR-Cas9 system was designed, as well as mosaicism and off-target cleavage at various sites, it was the first time CRISPR-Cas9 effectively cleaved endogenous genes on human embryos [104••]. It was proposed that dynamic guidelines, involving the wider society, should evolve with the progression of the scientific knowledge and should be established rather than imposing a moratorium on the advancement of such a promising technology [105]. This announcement was in keeping with a previous statement by the Hinxton Group, that concerns on possible human applications of these techniques should not inhibit the advancement of scientifically defensible basic research, especially as this very research will be the key to gaining such knowledge [106]. It did agree that human embryos or germ line cells subject to gene editing should not be used to establish pregnancies and produced a list of the current technical and moral issues, upon resolution of which, clinical somatic and germ line applications could be envisaged to eradicate devastating inheritable diseases [105]. Fear that one of CRISPR-Cas’ major advantages, its power for “democratization of gene targeting” [107] could be dangerous if used by the wrong people to “enhance” population minorities and reignite an interest for eugenics, maybe addressed by strong international jurisdictions and global public engagement [105, 106, 108]. However, before this could be performed, a deeper understanding of appropriate models (both cellular and animal, as well as human embryonic stem/iPS cell-derived germ line cells) to test efficacy and safety and multigenerational effects, and optimization of genome editing tools to minimize off-target events will be warranted. These include the development of more accurate and sensitive tools to assess off-target events and mosaicism [106]. Moreover, the types of cells and embryos that may be used in this context vary broadly and they need to be clarified to both the scientific and lay community to allow for constructive progression.

Non-viable preimplantation embryos from in vitro fertilization clinics may not satisfy the criterion of scientific validity, as the endogenous DNA repair mechanisms in their cells may be altered, but also raise ethical issues. Viable, unused embryos from these clinics would technically be more suitable; however, it could display high levels of mosaicism. Embryos specifically designed for research would be the most valuable in terms of research, but are the most ethically bound source of research material [106]. When considering such aspects, it should be kept in mind that human germ line editing may be the only solution to cure genetic diseases that manifest before birth and systemically (e.g., cystic fibrosis) in only one but very predominant tissue (e.g., muscular dystrophy) or in not easily accessible tissues (e.g., basal ganglia in Huntington’s disease). Patient-advocacy groups put hope in the use of these novel alternatives [109]. Concerns have further been expressed about the polygenic control of many human traits, the current difficulty to predict short-term effects of a genetic mutation and its side effects, as well as the apparent impossibility to anticipate its long-term effects in a putative future environment. To some, these points indicate that the hope to definitively eradicate human genetic diseases is only an illusion [110].

Unlike other moments in scientific history, the CRISPR-Cas system has opened an era of changes, which may span from groundbreaking therapeutic applications to daunting fears of irreversible perturbation of human evolution. It is essential that broad informed discussion across all exponents of society continue, to find a balance that will progressively lead to a society pivoted on the prioritization of human well-being and human rights.
